# The effects of anxiety on practice behaviors and performance quality in expert pianists

**DOI:** 10.3389/fpsyg.2023.1152900

**Published:** 2023-04-03

**Authors:** Edoardo Passarotto, Florian Worschech, Eckart Altenmüller

**Affiliations:** Institute of Music Physiology and Musicians' Medicine, University of Music, Drama and Media, Hannover, Germany

**Keywords:** practice behavior, anxiety, musical performance, professional musicians, playing-related injuries

## Abstract

**Introduction:**

During their career, musicians need to undergo intense periods of training to master musical instruments and become accomplished artists. Dysfunctional practice behaviors and anxiety are often mentioned among the possible risk factors for playing-related injuries in musicians. However, the mechanism through which these might lead to the onset of these injuries is still unclear. The present study aims at overcoming this limitation by investigating the relationship between quantitative measurements of anxiety, practice behaviors and music performance quality.

**Methods:**

The experiment consisted in monitoring practice behaviors in 30 pianists practicing a short musical task.

**Results:**

Most self-report anxiety measurements were positively correlated with practice time, especially those collected right before the practice sessions. Similar correlations were identified between anxiety and the number of repetitions of the musical task. Physiological markers of anxiety were only weakly related to practice behaviors. Subsequent analyses showed that high levels of anxiety were associated with poor quality of music performances at baseline. Nevertheless, the interaction between participants’ learning rate and anxiety measures showed no association with performance quality scores. Moreover, anxiety and performance quality co-developed during practice sessions, showing that pianists who improved their playing were also less anxious in the latter part of the experiment.

**Discussion:**

These findings suggest that anxious musicians are likely at higher risk of developing playing-related injuries related to overuse and repetitive strains. Future directions and clinical implications are discussed.

## 1. Introduction

Musicians are highly exposed to the risk of developing playing-related injuries. These are related to both genetic and environmental factors. Specifically, dysfunctional practice behaviors such as excessive repetitions and over-practice, anxious traits and stressful working conditions are often mentioned among their possible risk factors (Ackermann et al., 2012, 2014). However, the mechanism through which these might lead to the onset of playing-related injuries is still poorly understood.

### 1.1. The effects of practice and the role of practice quality

During their career, musicians need to undergo intense periods of training to master musical instruments and become accomplished artists. Not every musician progresses at the same pace: some students may in fact advance faster than other learning peers, despite similar practicing time (Bonneville-Roussy and Bouffard, 2015), which may result in higher academic proficiency and professional accomplishments. Several factors influence the long path to expertise in music: the literature on talent and giftedness suggests that in order to excel as musician it is often necessary to have proper tuition, financial support as well as motivation and natural abilities (Preckel et al., 2020).

Several studies have emphasized the importance of deliberate practice as it explains approximately 21% of variance in performance quality in music (Macnamara et al., 2014). Ericsson et al. (1993) specify that only a certain type of practice leads to proper improvements: they use the term deliberate practice to define “goal directed practice aimed at improving, requiring effort, concentration, determination and proper tuition” (Bonneville-Roussy and Bouffard, 2015, p.688).

Inefficient practice strategies may not only delay progress but also have secondary effects: low effectiveness may result in prolonged practice sessions, in the attempt to overcome the lack of improvement. Overpractice and repetitive practice behaviors may cause muscular overuse and lead to motor fatigue, increasing the risk of playing-related musculoskeletal disorders (Ackermann et al., 2012), which affect approximately 43% of all professional musician (Zaza, 1998). Playing-related injuries often consist of painful and disabling conditions with detrimental effects on musical performance and musicians’ career (Yoshimura and Chesky, 2009; Kenny and Ackermann, 2015).

Moreover, the literature suggests that long and demanding practice routines may have important effects on brain structures, as they may trigger dysfunctional plasticity and thus contribute to the onset of movement disorders as in the case of musicians’ focal dystonia (Altenmüller and Jabusch, 2009). This framework is further supported by evidence from animal models where symptoms and neural conditions comparable to focal hand dystonia have been successfully induced in primates by means of massed repetitions (Byl et al., 1997; Byl, 2007).

### 1.2. Anxiety in musicians

Music performance anxiety is often mentioned among the risk factors of playing-related injuries in music and it affects between 16.5 and 60% of all musicians (Fernholz et al., 2019). It is a multidimensional construct and it manifests itself on both cognitive and somatic dimensions (Miller and Chesky, 2004; Papageorgi et al., 2007; Papageorgi, 2022), including physiological symptoms and behavioral changes as increased heart rate, reduced heart rate variability (LeBlanc et al., 1997), shaky and numb fingers as well as arm and neck stiffness. It can also involve psychological reactions as exaggerated fear and apprehension as well as cognitive impairments as lack of concentration and memory slips (Kenny, 2011).

Anxiety is considered a comorbidity of performance-related musculoskeletal disorders (Ackermann et al., 2012; Kenny and Ackermann, 2015). For instance, Ranelli et al. (2015) showed significant associations between music performance anxiety and playing-related pain in children, during early stages of learning a musical instrument. Moreover, trait anxiety is listed among the psychological trigger and risk factors of musicians’ focal dystonia: Altenmüller and Jabusch (2009) showed that musicians affected by this movement disorder have higher levels of anxiety, which might lead to repetitive practice behaviors and stress-induced consolidation of dystonic movements. The literature suggests that a common response to anxiety may include ritualistic behaviors, rigidity as well as repetitive motor patterns (Lang et al., 2015): anxious individuals seem to frequently perform familiar tasks in order to reestablish a feeling of order and control. Sporn et al. (2020) supported this idea, showing that state anxiety may reduce motor exploration during the acquisition of new motor tasks, thus affecting the quality of learning. However, the consistency of these findings in the context of musical practice is still to be assessed.

While the effect of anxiety on performance quality is rather controversial (Brotons, 1994; Cohen and Bodner, 2019), the relationship between anxiety and practice behaviors in music remains almost undocumented. A pioneering study by McPherson and McCormick (1999) analyzed practice behaviors, anxiety and other psychological traits in 190 pianists preparing for performance examinations in music academies. Their results indicated a significant positive association between pre-performance anxiety and the amount of weekly practice in the month preceding the examinations. Moreover, musicians who incorporated more technical exercises in their practice routines exhibited higher levels of music performance anxiety. Nonetheless, the study did not clarify why anxiety might increase the amount of practice nor did it examine the relationship between anxiety and practice behaviors.

### 1.3. Aim of the study

The present study aims at investigating the relationship between anxiety, practice behaviors, and performance quality by monitoring 30 pianists practicing a short musical excerpt. Specifically, it investigates whether musicians who show high levels of anxiety practice longer, employ more repetitions and improve at a slower pace than their less anxious peers.

## 2. Methods

### 2.1. Design

The study involved quantitative measurements of anxiety, practice behaviors, and performance quality with the aim at investigating the relationship between the three variables. The experiment consisted in monitoring practice behaviors in healthy young pianists while practicing a short musical task: testing healthy musicians allowed to avoid biases related to playing-related injuries, their time course, and treatments. To measure improvements in performance quality, run-throughs of the musical task were recorded at baseline and acquisition, before and after the practice sessions. During the experiment, anxiety was assessed by means of self-report measurements as well as physiological data. The testing procedure was inspired and freely adapted from a previous study by Bangert et al. (2014).

The Central Ethics Committee at Leibniz University Hannover approved the present study.

### 2.2. Participants

Participation was open to student pianists from the University of Music, Drama, and Media in Hannover, Germany. Participants were clinically healthy and did not report any pain or injury at the time of the experiment. Moreover, they did not have any previous experience with the musical task used in the experiment, nor with the musical piece on which it was based. 33 musicians took part in the experiment: three participants were excluded from the study for not following the instructions provided by the experimenter.

The resulting sample (*N* = 30) had a mean age of 24.13 years (SD = 3.92), 60% were females and 40% were males. 43.3% of the sample were undergraduate students while the remaining 56.7% were enrolled in postgraduate study courses, as Master of Music or Konzertexamen. Further information is reported in Table 1. All participants were above 18 years of age, and they received a compensation of 50€ for their collaboration with the investigation.

**Table 1:** Descriptive statistics for the variables age, age at which participants started playing the piano, and lifetime practice.

	Mean	SD
Age	24.13	3.92
Age at which participants started playing piano	6.50	3.63
Lifetime practice (hours)	17,723	10,273

*N* = 30.

### 2.3. Materials and instruments

#### 2.3.1. Baseline measurements

At the beginning of the experiment, participants completed a questionnaire investigating their demographics, the current degree program and the amount of lifetime practice, measured in hours (see Table 1). The survey also included the Spielberger State–Trait Anxiety Inventory (STAI, Spielberger, 1989) as well as six items aimed at investigating participants’ history of playing-related injuries, to assess their eligibility for participating in the study.

#### 2.3.2. Piano performance

**Figure 1:**
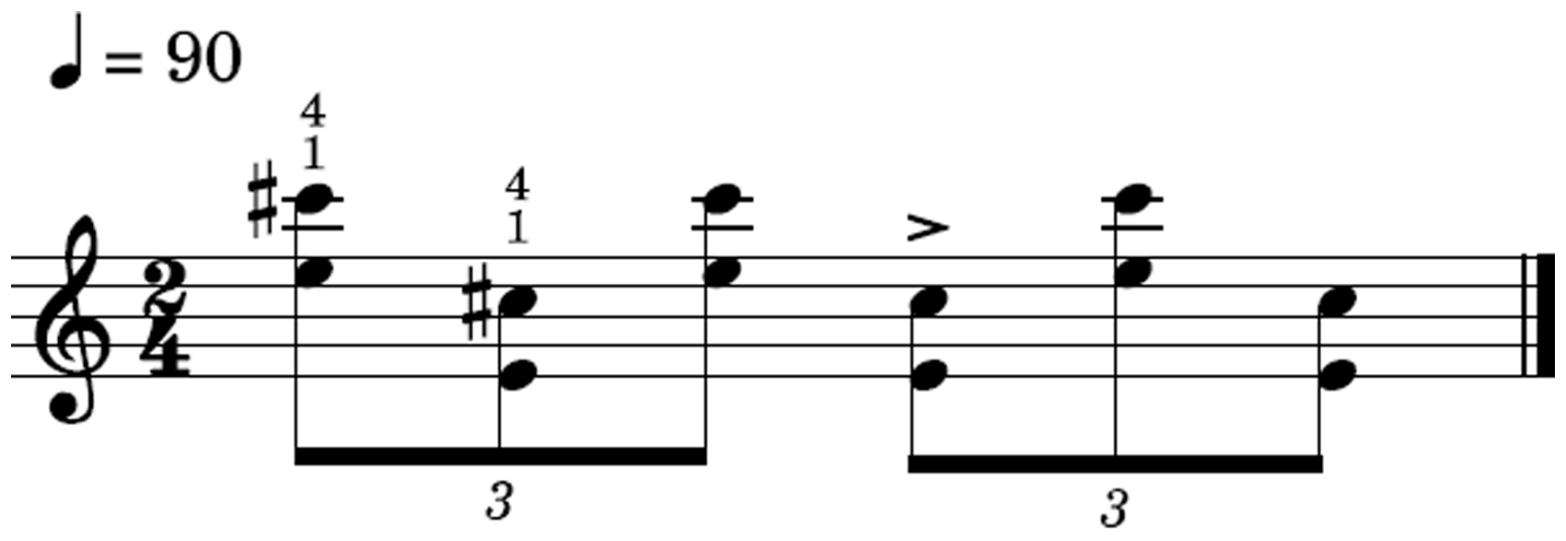
Musical task inspired by Scriabin, Sonata op.53.

The musical task used in the experiment was inspired by Scriabin’s Sonata op.53, bar 85, and consisted of multiple bidirectional octave leaps in a simple rhythmic structure performed with their right hand only, as shown in Figure 1. During baseline and acquisition assessment phases the excerpt was repeated five times: participants were allowed to take breaks of few seconds (M = 2.21 s, SD = 1.13 s) in between repetitions. Both tests were assisted by a metronome, set at 90 beats per minute (bpm). Piano performances were recorded using a CASIO PX-730 electric piano, which was connected to a MOTU 828mk3 soundcard *via* MIDI interface.

Performance quality was assessed based on four parameters: *wrong notes, missed notes, rhythmic accuracy,* and *loudness homogeneity. Wrong notes* measured the number of notes extraneous to the excerpt while *missed notes* represent those that were not played by the participant. Given the homorhythmic texture and regularity in dynamics of the musical task, *rhythmic accuracy* and *loudness homogeneity* were assessed as standard deviations of inter-onset intervals and MIDI keystroke velocity, respectively. The four parameters were computed across all five repetitions of the musical task performed during each assessment phase. Baseline and acquisition performances were evaluated separately for each participant.

#### 2.3.3. Practice behaviors

Practice behaviors during the practice session were analyzed according to the following parameters: *practice time,* measuring the duration of individual practice sessions in minutes, *keystrokes on target*, defined as the total number of piano keystrokes on pitches belonging to the musical task (see Figure 1), and *total keystrokes,* indicating the total number of keystrokes during the practice session irrespective of the pitch.

#### 2.3.4. Anxiety measures

**Figure 2:**
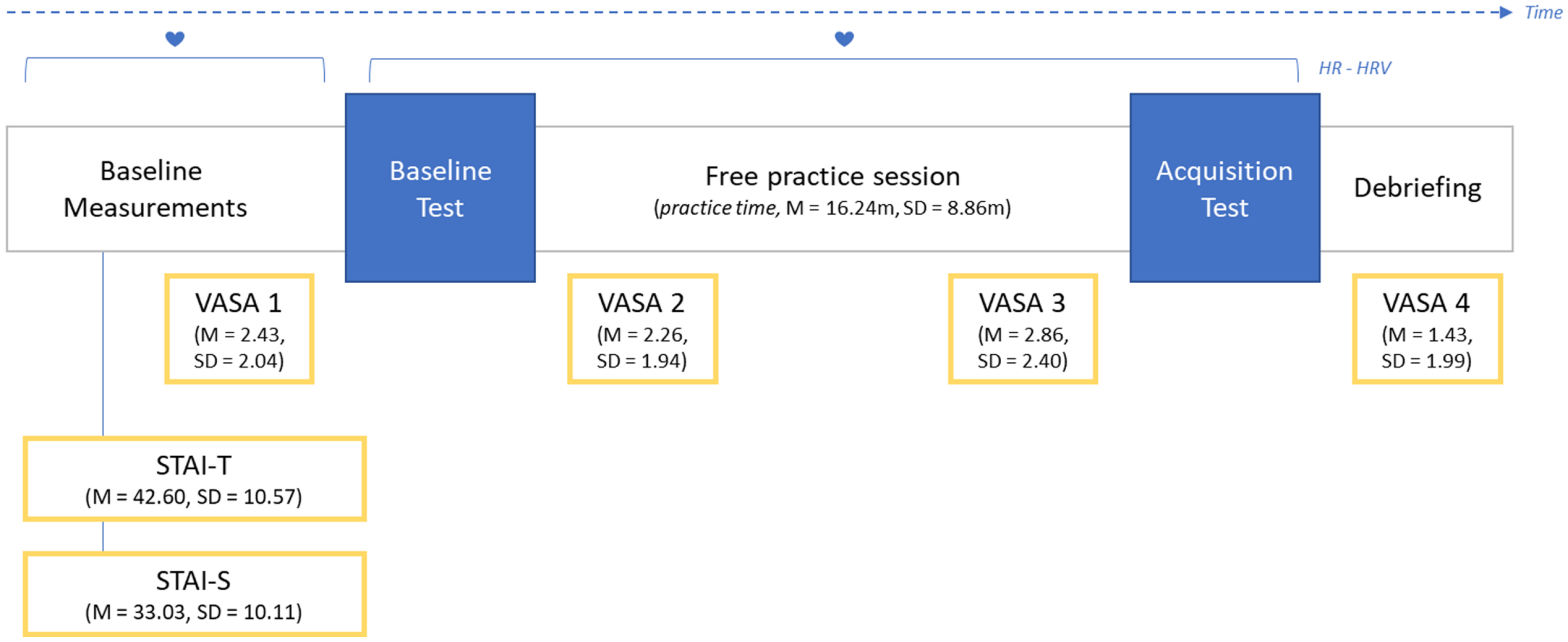
Timeline of the experimental procedure and descriptive statistics for anxiety measures and practice time. Anxiety was assessed by the Spielberger State–Trait Anxiety Inventory (STAI), measuring Trait (STAI-T) and State anxiety (STAI-S), Visual Analogue Scales for Anxiety (VASA) at four timepoints as well as continuous electrocardiogram.

During the experiment, anxiety was assessed by means of self-report measurements as well as physiological data. State and trait anxiety were measured through the Spielberger State–Trait Anxiety Inventory (STAI, Spielberger, 1989): the measurement instrument consists of 40 items in total, rated on a four-point Likert scale of agreement, aimed at assessing state anxiety (STAI-S, 20 items) and trait anxiety (STAI-T, 20 items) separately. In addition to this, Visual Analogue Scales of Anxiety (VASA) were implemented to monitor participants’ state anxiety throughout the experimental procedure: they consisted in a single item investigating how anxious and tense participants felt right before and after each performance assessment phase. VASA was rated on an 11-point ordinal scale, with values ranging between 0, “not at all,” and 10, “very much.” As shown in Figure 2, VASA 1 and VASA 3 measured pre-performance anxiety, before baseline and acquisition assessment procedures, respectively. VASA 2 and VASA 4 quantified post-performance anxiety after the two tests. The placement, labeling and descriptive statistics of the anxiety measurements used in the study is reported in Figure 2.

Participants’ heart activity was monitored through electrocardiograms (ECG). The resulting data were used to compute mean Heart Rate (*HR*) and the Coefficient of Variation of RR intervals (*CVRR*; *see* Sporn et al., 2020; Hein et al., 2021), as explained in the section Data processing. The recordings were performed using HEI ECG-AMP04 sensors placed in a three-lead ECG configuration and connected to a CED Micro1401-3 data acquisition unit. ECG signal was recorded by Signal 5.12 data acquisition software, sampling data at 1000 Hz. ECG and piano performance data were synchronized by means of analogue pulse signals, allowing a synchronization accuracy greater than one millisecond per minute of recording. MIDI data and pulse signals were recorded through Reaper v6.36 digital audio workstation, sampling data at 44,100 Hz and 32 bits.

### 2.4. Testing procedure

Figure 2 represents the timeline of the experimental procedure. The experiment took place in a quiet room of approximately 9 m^2^. At their arrival, participants filled out a questionnaire investigating their musical background, history of playing-related injuries as well as state and trait anxiety (STAI-S and STAI-T). Subsequently, the experimenter applied the ECG electrodes on participants’ chest and invited them to freely warm-up on the MIDI piano used for the test, to familiarize with the musical instrument. After warm-up, the Principal Investigator (PI) explained the experimental procedure: participants were asked to freely practice a short musical task. Their goal was to play it as accurately as possible in terms of wrong and missed notes, rhythmical precision, and loudness regularity. No time constraints were imposed: participants were allowed to practice the musical excerpt as much as they wanted, using the practice strategies they preferred. During the practice session, the PI left the experiment room to reproduce conditions comparable with solitary practice. Participants were instructed to call back the researcher at the end of their practice session *via* phone.

Performance quality was assessed at baseline and acquisition, before and after the practice sessions, and each test consisted of five repetitions of the musical task, assisted by a metronome, set at 90 bpm. Participants were not allowed to practice the musical excerpt before the baseline performance quality assessment. However, they could analyze its notation and listen to it through a dedicated audio recording. To induce anxiety, participants were informed at the beginning of the experiment that their performances were going to be video recorded and rated by three professors of music at the local university. During the entire experiment, MIDI and ECG recordings were used to monitor participants’ behavior and heart activity, respectively.

Note that the testing procedure here reported is part of a longer set of measurements and tests whose results will be described in future reports.

### 2.5. Data processing

Electrocardiogram signal was visually inspected to manually reject artifacts. Subsequently, a 30 Hz low-pass filter was applied, and R peaks were identified from QRS complexes using the findpeaks function from the R-package pracma (Borchers, 2022). The R-package RHRV (Rodriguez-Linares et al., 2022) allowed to additionally filter the resulting data by rejecting datapoints indicating unacceptable physiological values (i.e., outliers with HR < 25 bpm and HR > 200 bpm). Finally, the same R-package was used to interpolate data at 4 Hz. Thus, mean Heart Rate (*HR*) and the Coefficient of Variation of RR intervals (*CVRR*; *see* Sporn et al., 2020; Hein et al., 2021) were computed from the pre-processed data. To quantify increases or decreases in HR during the experiment, linear regression models were used to linearly predict HR by time of the measurement for each participant: thus, individual slope coefficients (*slope HR*) were extracted from regression models and used for the analyses. All physiological parameters were measured during practice sessions and performance evaluation phases.

MIDI data were analyzed through a computerized assessment procedure coded in R-language. Detailed information about the four performance quality parameters is reported in Appendix 1.

Subsequently, a principal component analysis procedure was performed with the R-package *lavaan* (Rosseel et al., 2022) to obtain an aggregate measure of performance quality, referred to as *performance quality* scores: three of four performance quality parameters loaded adequately on a single factor (eigenvalue = 1.78), with factor loading ranging from 0.58 to 0.86. *Rhythmic accuracy* did not load sufficiently well on the latent variable probably due to ceiling effects in the measurement (see Appendix 1). Therefore, it was discarded from the analyses. Note that the resulting performance quality scale is an inverted scale: low *performance quality* scores correspond to high performance quality levels and vice versa. Finally, MIDI recordings were used to compute the variable *time*, which indicates at what timepoint in the experiment each performance was recorded, taking individual baseline performances as a reference, when *time* = 0.

### 2.6. Data analyses

All participants completed the experiment in its entirety and no missing data was produced. Correlation matrices were used to investigate the relationship between anxiety and practice behaviors, considering both self-report and physiological measures of anxiety as well as the practice behaviors descriptors mentioned in the previous paragraphs. As shown in Appendix 2, no significant differences in anxiety measures were found between female and male pianists (*p* > 0.05). Therefore, *gender* was not considered in the analyses.

The effect of anxiety and *time* on *performance quality* was assessed *via* Bayesian mixed effects regression models for repeated measures analyses. The models entered baseline and acquisition *performance quality* scores as criterion, *time* and anxiety measures as fixed effects and random intercepts per *performer* with random slopes per *time* as random effects. Note that baseline *performance quality* scores corresponded to *time* = 0 (for further information, see the Data processing section). Therefore, the main effects of stationary regressors (i.e., anxiety measures) described their relationship with baseline *performance quality* scores. The main effect of *time* quantified participants’ learning rate, namely the average improvement in performance quality per minute of practice. Interactions between *time* and anxiety measures assessed differences in learning rate related to different anxiety levels. The analyses were run considering all the anxiety measures mentioned in the previous paragraphs as predictors: only the most relevant findings are reported in the present manuscript.

In this study, Bayesian effect estimates are reported along with 95% Credible Intervals (CI) in squared brackets. Thus, if this interval does not contain zero, the regressors are assumed to exert a (positive or negative) effect on the dependent variable with a probability of at least 95% (Hespanhol et al., 2019).

Latent change score models were modeled *via* the R-package *lavaan* (Rosseel et al., 2022) and used to analyze the relationship between self-reported anxiety, performance quality, and the development of these parameters during the experiment. The model measured changes in performance quality, pre-performance anxiety (VASA 1 and VASA 3) and post-performance anxiety (VASA 2 and VASA 4) due to practice and their covariance. Subsequently, it investigated correlations and cross-correlations between change scores and baseline values. The model was run under maximum likelihood estimation and its fit was evaluated in terms of χ^2^, CFI, TFI, RFI, and SRMR values. RMSEA were not considered in the analyses, due to their limited validity in models with small degrees of freedom and sample sizes (Kenny et al., 2015). For a comprehensive overview of latent change score models, see Kievit et al. (2018).

All statistical analyses were conducted using the software RStudio (RStudio Team, 2021).

## 3. Results

### 3.1. The relationship between anxiety and practice behaviors

As shown in Table 2, *practice time*, *keystrokes on target*, and *total keystrokes* were positively correlated with most self-report anxiety measures, particularly with VASA 1 and VASA 2, at *p* < 0.05. Physiological markers of anxiety were only weakly and non-significantly correlated to practice behaviors parameters with the only exception of *mean HR,* which was positively correlated with *total keystrokes*, *r*(28) = 0.395, *p* = 0.03.

**Table 2:** Correlations between anxiety measures and practice behavior descriptors.

	Practice time	Total keystrokes	Keystrokes on target
STAI-T	0.224	0.352	0.180
STAI-S	0.216	0.362^*^	0.234
VASA 1	0.408^*^	0.470^*^	0.295
VASA 2	0.472^*^	0.438^*^	0.379^*^
VASA 3	0.219	0.191	0.236
VASA 4	0.062	0.051	0.076
mean HR	0.310	0.395^*^	0.314
CVRR	−0.095	−0.199	−0.114
slope HR	−0.195	−0.209	−0.199

*N* = 30; ^*^correlations are significant at *p* < 0.05. The table reports Pearson’s *r* coefficients.

Thus, high levels of anxiety immediately before and after the baseline assessment procedure were associated with longer practice sessions and more repetitions.

### 3.2. The effect of anxiety and time on performance quality

Bayesian mixed effects regression models were run to investigate the effect of anxiety measures and *time* on performance quality scores during the experiment. All anxiety measurers were standardized across participants. Table 3 report the summary of the final models, where performance quality scores were predicted by either VASA 1 (model 1) or VASA 2 (model 2). *Time*, VASA 1, and VASA 2 had meaningful main effects on performance quality scores, but there were not relevant interactions between *time* and anxiety measures. These results did not generalize to the other anxiety variables included in the study, probably due to their lower temporal specificity and relevance. The two models reported in Table 3 explained between 43 and 50% of the variance in *performance quality* scores. Nevertheless, we prefer not to further comment on *R*^2^ values, as their interpretation is quite controversial (i.e., Ozili, 2022), nor is a comparison between the two models reported in Table 3 meaningful, as they consider participants’ state anxiety measured at two distinct timepoints.

**Table 3:** The effect of state anxiety (VASA 1 and VASA 2) and time on performance quality scores.

	Model 1	Model 2
Fixed Effects	Estimate [95% CI]	Estimate [95% CI]
Intercept	0.40 [0.10, 0.72]	0.42 [0.14, 0.73]
Time	−0.05 [−0.08, −0.03]	−0.05 [−0.08, −0.03]
VASA 1^a^	0.33 [0.01, 0.67]	–
Time: VASA 1^a^	0.00 [−0.03, 0.02]	–
VASA 2^a^	–	0.48 [0.20, 0.76]
Time: VASA 2^a^	–	−0.00 [−0.03, 0.02]
Random Effects		
Performer:		
Intercept	0.42 [0.23, 0.59]	0.40 [0.23, 0.57]
Time	0.02 [0.00, 0.03]	0.02 [0.00, 0.03]
cor(Intercept, Time)	−0.06 [−0.73, 0.68]	−0.13 [−0.76, 0.60]
residuals	0.72 [0.53, 0.94]	0.69 [0.51, 0.89]
Coefficients of determination		
Conditional *R*^2^	0.43 [0.26, 0.60]	0.50 [0.35, 0.66]
Marginal *R*^2^	0.31 [0.15, 0.44]	0.39 [0.24, 0.51]

*N* = 30; ^a^VASA 1 and VASA 2 were standardized across participants. The table reports fixed and random effects followed by 95% Credible Intervals (CI) in square brackets []. Performance quality scores were predicted by either VASA 1 (model 1) or VASA 2 (model2). Time quantified participants’ rate of learning, namely the average improvement in performance quality per minute of practice. Interactions between time and anxiety measures assessed differences in learning rate related to different anxiety levels. For further information about the model, see the dedicated Data analyses section.

In summary, high levels of anxiety right before and after the baseline performance assessment were associated with poor *performance quality* at baseline. Nevertheless, the interaction between learning rate (*time*) and anxiety measures showed no association with *performance quality* scores.

### 3.3. Co-development of anxiety and performance quality measures

**Figure 3:**
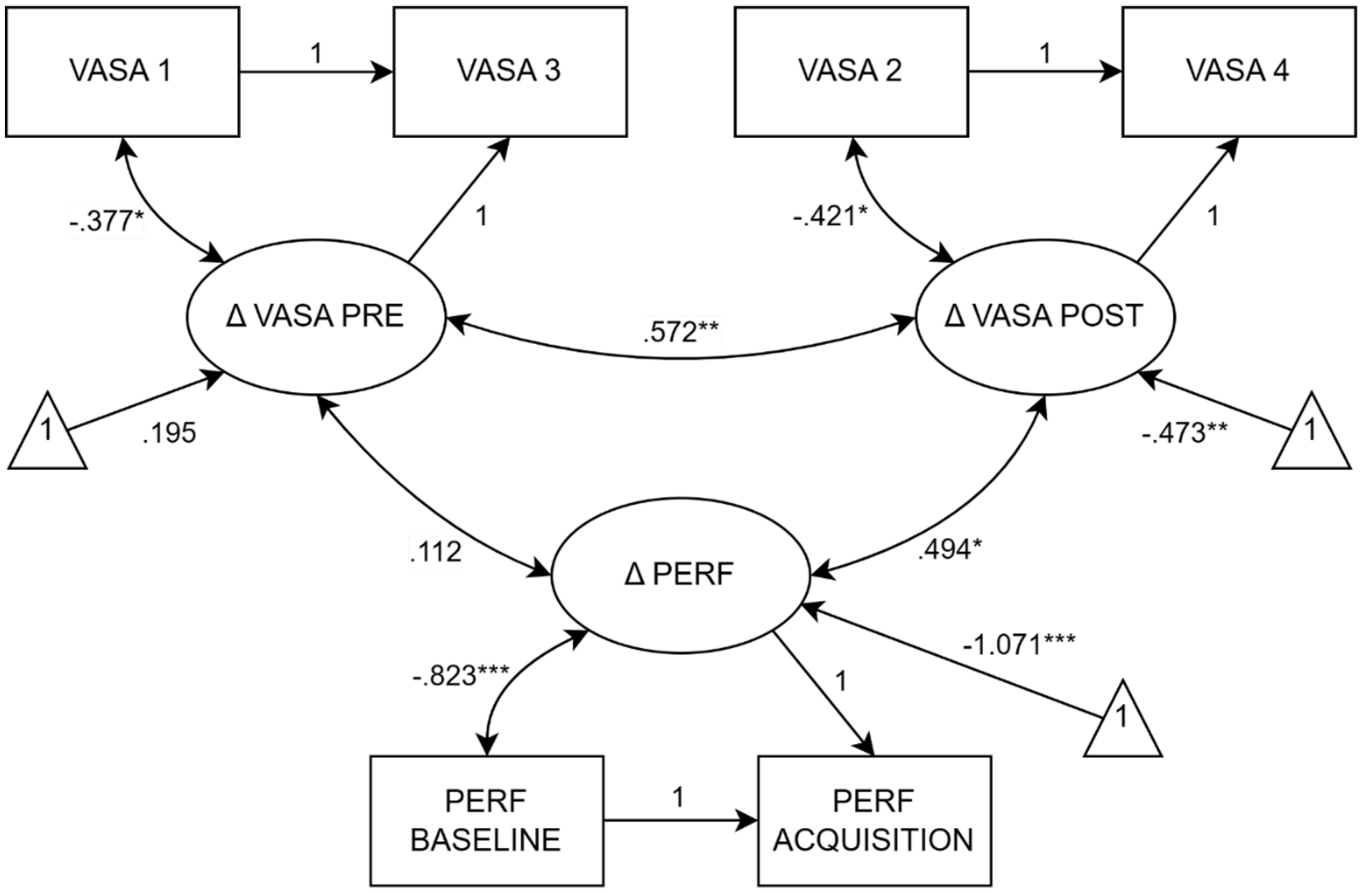
Co-development of anxiety and performance quality measures. *N* = 30; ^*^covariances are significant at *p* < 0.05; ^**^covariances are significant at *p* < 0.01; ^***^covariances are significant at *p* < 0.001. PERF, *performance quality*; VASA, visual analogue scales of anxiety. Latent change score model: only the most relevant covariances are reported. For further information, see the Data analyses section and Appendix 3.

Figure 3 represents the latent change score model which was used to investigate the development of performance quality as well as pre-and post-performance anxiety during the experiment. The model showed good fit indices [χ^2^(1, 21) = 0.129, *p* > 0.05, CFI = 1.000, TLI = 1.000, RFI = 0.972, SRMR = 0.017] and indicated a significant positive correlation between pre-and post-performance anxiety change scores, *r* = 0.572, *p* = 0.006. Improvements in *performance quality* scores were moderately correlated with post-performance anxiety change scores only, *r* = 0.494, *p* = 0.014.

Moreover, two significant correlations between baseline scores and one cross-correlation between baseline and change scores were identified, evidencing the close relationship between anxiety and performance measures: VASA 2 was positively correlated with VASA 1, *r* = 0.562, *p* = 0.007, and baseline performance, *r* = 0.483, *p* = 0.017. Post-performance anxiety change scores were negatively related to baseline performance, *r* = −0.454, *p* = 0.023. Thus, performance quality scores and anxiety measurements seemed highly related and developed within a complex structure of mutual influences. For further information, see Appendix 3.

## 4. Discussion

The present study aimed at investigating the relationship between anxiety and practice behaviors in a sample of student pianists practicing a short musical task inspired by the piano literature. Specifically, it addressed the question whether musicians who show high levels of anxiety practice longer, employ more repetitions and improve at a lower rate than their colleagues.

### 4.1. Summary of results

The results indicated that most self-report anxiety measurements were positively correlated with practice time, especially those collected right before the practice sessions (see Table 2). Similar correlations were identified between anxiety and the number of repetitions of the musical task. Physiological markers of anxiety were only weakly related to practice behaviors’ descriptors except for mean heart rate, which was significantly and positively correlated with the total number of keystrokes recorded during the practice sessions. Subsequent analyses showed that anxiety was associated with poor performance quality. Nevertheless, the interaction between participants’ learning rate and anxiety measures showed no association with performance quality scores. Finally, anxiety and performance quality co-developed during the practice sessions, showing that pianists who improved their playing were also less anxious in the latter part of the experiment.

### 4.2. Anxiety, performance, and practice

In the present study, state anxiety was associated with longer practice sessions and repetitive practice behaviors, probably due to its negative effects on performance quality, in line with the literature (McPherson and McCormick, 1999; Yoshie et al., 2009a,b). These findings are important as they support the hypothesis that anxious musicians are at higher risk of developing occupational diseases as a result of overuse and repetitive strain (Altenmüller et al., 2015; Kenny and Ackermann, 2015).

Pre-and post-performance anxiety were closely related to piano performance, as they seem to be an emotional anticipation and response to poor performance quality. The effect of anxiety on performance quality is controversial and findings in the literature are rather inconsistent (Brotons, 1994; Ioannou et al., 2016; Cohen and Bodner, 2019). This might be explained by two methodological issues here avoided: first, the present study evaluated performance quality through an objective computerized assessment procedure, avoiding human judgments and their low reliability (Thompson and Williamon, 2003; Passarotto et al., in press). Furthermore, anxiety was measured at multiple time points and by different approaches, investigating both participants’ subjective anxiety and physiological response throughout the experiment. This allowed to account for the high temporal variability in anxiety measurements (Rossi and Pourtois, 2012). Less reliable and less time-specific measurements (i.e., assessing only trait anxiety) might reduce the sensitivity of the analyses and lead to different results.

A plausible explanation for the relationship and co-development of anxiety and performance quality comes from the self-efficacy theory (Bandura, 1997): previous findings suggest that musicians who believe they have the necessary resources to achieve their goals are less anxious and perform better than their colleagues (McPherson and McCormick, 2006; González et al., 2018). In the short timeframe considered in this study, repetitive behaviors and improvements might have helped participants to increase their self-confidence while reducing anxiety. Thus, participants who were more anxious at the beginning of the practice session performed poorly at baseline and needed more time and repetitions to achieve a satisfactory performance quality. Nevertheless, the present study did not include any measure of self-efficacy and further studies are needed to verify this hypothesis.

### 4.3. Limitations

The present study comes with several limitations. First, it was conducted on a small sample of piano students and the findings might not generalize to other musical instruments or levels of expertise. Moreover, participants practiced a very short musical excerpt only few seconds in length which might not be representative of longer and more articulated musical structures.

The association between anxiety and repetitive practice behaviors here reported was identified on a practice task with a rather repetitive musical structure. This might have discouraged participants from showing greater variability in practice strategies, influencing the outcome of the analyses. Furthermore, the length of the experiment was often too short to analyze ECG signals appropriately by means of more informative approaches (i.e., spectral analysis). Anxiety was measured *via* well-established self-report measurement instruments which, however, were not specifically designed for musicians. The study investigated the effect of anxiety on performance and practice only at the early stages of learning a new musical excerpt which might not apply to later learning phases and highly trained repertoires. Finally, the relationship between anxiety and performance quality was investigated without considering the contribution of other covariates related to motor learning as perceptual and cognitive abilities (Anderson et al., 2021) as well as biomechanical characteristics of pianists’ hands (Yoshimura et al., 2006; Yoshimura and Chesky, 2009).

### 4.4. Future development

The experimental procedure here implemented seems well suited for research projects investigating motor learning and practice behaviors in music, as already demonstrated by its original authors (Bangert et al., 2014). Testing healthy musicians allowed to avoid biases related to playing-related injuries, their time course and treatment. Nevertheless, future studies should assess the consistency of the present findings in different cohorts of musicians, especially in samples of musicians suffering from overuse and repetitive strain injuries. They could also evaluate the effectiveness of interventions aimed at reducing performance anxiety in terms of performance quality, practice time and practice behaviors. Finally, future studies might investigate the effect of anxiety on retention of knowledge and memory consolidation in music, implementing longitudinal study designs.

## 5. Conclusion

In conclusion, this is the first study to systematically investigate the process through which anxiety interacts with practice behaviors: it provided a plausible and rational framework explaining the role of anxiety and practice behaviors in triggering playing-related injuries in musicians, for which they are rightfully considered risk factors. The results here reported highlight the importance of training protocols specifically aimed at improving practice effectiveness and reducing music performance anxiety, therefore preventing playing-related injuries in musicians.

## Data availability statement

The raw data supporting the conclusions of this article will be made available by the authors, without undue reservation.

## Ethics statement

The studies involving human participants were reviewed and approved by Central Ethics Committee at Leibniz University Hannover. The patients/participants provided their written informed consent to participate in this study.

## Author contributions

EP and EA contributed to conception and design of the study. EP collected the data. EP and FW performed the statistical analysis. EP wrote the manuscript. All authors contributed to the article and approved the submitted version.

## Conflict of interest

The authors declare that the research was conducted in the absence of any commercial or financial relationships that could be construed as a potential conflict of interest.

## Publisher’s note

All claims expressed in this article are solely those of the authors and do not necessarily represent those of their affiliated organizations, or those of the publisher, the editors and the reviewers. Any product that may be evaluated in this article, or claim that may be made by its manufacturer, is not guaranteed or endorsed by the publisher.
