# Supplementary material for: The effects of anxiety on practice behaviors and performance quality in expert pianists
Source: Front Psychol. 2023 Apr 3;14:1152900. doi: 10.3389/fpsyg.2023.1152900 (PMC10106594; doi:10.3389/fpsyg.2023.1152900)
Supplement: Supplementary file 1 [file Table_1.pdf]

APPENDIX

Appendix 1. Performance quality descriptors.

	wrong notes	missed notes	rhythmic accuracy	loudness homogeneity
Baseline test				
Mean	8.633	9.600	0.018	16.233
SD	6.094	6.095	0.007	3.593
Acquisition test				
Mean	3.500***	2.433***	0.016	13.592**
SD	3.093	2.674	0.007	3.843

Notes: N = 30; **significant improvement from baseline, paired samples t-test significant at $p < .01$; *** significant improvement from baseline, paired samples t-test significant at $p < .001$. *Wrong notes* measures the number of notes extraneous to the musical task while *missed notes* measured those that were not played by the performer. *Rhythmic accuracy* and *loudness homogeneity* were assessed as standard deviations of inter onset intervals (reported in seconds) and MIDI keystroke velocity values respectively. The four parameters were computed across all five repetitions of the musical task performed during each assessment phase.

Appendix 2. Descriptive statistics for anxiety scores from female (N = 18) and male (N = 12) pianists and from the complete sample (N = 30).

	Females		Males		Overall	
	Mean	SD	Mean	SD	Mean	SD
STAI-T	43.33	10.24	41.50	11.41	42.60	10.57
STAI-S	32.00	8.39	34.58	12.52	33.03	10.11
VASA 1	2.17	1.72	2.83	2.48	2.43	2.05
VASA 2	2.22	1.66	2.33	2.39	2.27	1.95
VASA 3	3.17	2.71	2.42	1.88	2.87	2.40
VASA 4	1.56	1.92	1.25	2.18	1.43	1.99
mean HR	92.85	11.68	86.41	14.43	90.68	14.38
CVRR	7.83	1.89	8.61	3.33	7.85	2.47
slope HR	0.01	0.03	0.01	0.01	0.01	0.02

Notes: no significant differences were found between female and male pianists ($p > .05$).

Appendix 3. Covariance between anxiety and performance quality scores and change scores.

Covariances	Estimate	SE	z-value	*p*	Std.lv	Std.all
delta_perf ~~						
perf_baseline	-0.847	0.241	-3.521	0.001	-0.798	-0.823
VASA_pre ~~						
VASA_1	-1.688	0.869	-1.944	0.052	-0.759	-0.377
VASA_post ~~						
VASA_2	-1.410	0.636	-2.218	0.027	-0.801	-0.421
delta_perf ~~						
VASA_pre	0.264	0.403	0.655	0.512	0.112	0.112
VASA_post	0.924	0.375	2.464	0.014	0.494	0.494
VASA_pre ~~						
VASA_post	2.241	0.810	2.765	0.006	0.572	0.572
VASA_1 ~~						
perf_baseline	0.381	0.214	1.782	0.075	0.381	0.195
VASA_2 ~~						
perf_baseline	0.799	0.328	2.440	0.015	0.799	0.434
VASA_1	2.105	0.787	2.676	0.007	2.105	0.551
delta_perf ~~						
VASA_2	-0.371	0.315	-1.178	0.239	-0.350	-0.184
VASA_pre ~~						
perf_baseline	-0.324	0.376	-0.862	0.389	-0.146	-0.150
VASA_2	-0.579	0.773	-0.749	0.454	-0.260	-0.137
VASA_post ~~						
perf_baseline	-0.772	0.332	-2.323	0.020	-0.438	-0.453
VASA_1	-0.691	0.576	-1.200	0.230	-0.393	-0.195

Notes: N = 30. Latent change score model: it was used to investigate the development of performance quality as well as pre- and post-performance anxiety during the experiment. Perf = performance quality, VASA = Visual Analogue Scales of Anxiety.
